# Relevance of Aβ42/40 Ratio for Detection of Alzheimer Disease Pathology in Clinical Routine: The PLM_R_ Scale

**DOI:** 10.3389/fnagi.2018.00138

**Published:** 2018-05-28

**Authors:** Sylvain Lehmann, Constance Delaby, Guilaine Boursier, Cindy Catteau, Nelly Ginestet, Laurent Tiers, Aleksandra Maceski, Sophie Navucet, Claire Paquet, Julien Dumurgier, Eugeen Vanmechelen, Hugo Vanderstichele, Audrey Gabelle

**Affiliations:** ^1^Laboratoire de Biochimie Protéomique Clinique, Institute of Regenerative Medicine and Biotherapies, Centre Hospitalier Universitaire de Montpellier, Montpellier, France; ^2^Université de Montpellier, Montpellier, France; ^3^Département de Neurologie, Centre Mémoire de Ressources et de Recherche de Montpellier, Montpellier University Hospital, Centre Hospitalier Universitaire de Montpellier, Montpellier, France; ^4^Groupe Hospitalier Lariboisière Fernand-Widal, INSERM U942, Centre de Neurologie Cognitive, Université Paris Diderot, Paris, France; ^5^ADx NeuroSciences, Ghent, Belgium

**Keywords:** Alzheimer’s disease, biomarkers, cerebrospinal fluid (CSF), screening scale

## Abstract

**Background:** Cerebrospinal fluid (CSF) biomarkers (Aβ peptides and tau proteins) improved the diagnosis of Alzheimer’s disease (AD) in research and clinical settings. We previously described the PLM-scale (Paris-Lille-Montpellier study), which combines Aβ42, tau, and phosphorylated ptau(181) biomarkers in an easy to use and clinically relevant way. The purpose of this work is to evaluate an optimized PLM_R-_scale (PLM ratio scale) that now includes the Aβ42/Aβ40 ratio to detect AD versus non-AD (NAD) participants in clinical routine of memory centers.

**Methods:** Both scales were compared using 904 participants with cognitive impairment recruited from two independent cohorts (Mtp-1 and Mtp-2). The CSF Aβ42/Aβ40 ratio was measured systematically in Mtp-1, and only on biologically discordant cases in Mtp-2. Two different ELISA kit providers were also employed. The distribution of AD and NAD patients and the discrepancies of biomarker profiles were computed. Receiver Operating Characteristic curves were used to represent clinical sensitivity and specificity for AD detection. The classification of patients with the net reclassification index (NRI) was also evaluated.

**Results:** Nine hundred and four participants (342 AD and 562 NAD) were studied; 400 in Mtp-1 and 504 in Mtp-2. For AD patients, the mean CSF Aβ42 and CSF Aβ42/40 ratio was 553 ± 216 pg/mL and 0.069 ± 0.022 pg/mL in Mtp-1 and 702 ± 335 pg/mL and 0.045 ± 0.020 pg/mL in Mtp-2. The distribution of AD and NAD differed between the PLM and the PLM_R_ scales (*p* < 0.0001). The percentage AD well-classified (class 3) increased with PLM_R_ from 38 to 83% in Mpt-1 and from 33 to 53% in Mpt-2. A sharp reduction of the discordant profiles going from 34 to 16.3% and from 37.5 to 19.8%, for Mtp-1 and Mtp-2 respectively, was also observed. The AUC of the PLM_R_ scale was 0.94 in Mtp-1 and 0.87 in Mtp-2. In both cohorts, the PLM_R_ outperformed CSF Aβ42 or Aβ42/40 ratio. The diagnostic performance was improved with the PLM_R_ with an NRI equal to 44.3% in Mtp-1 and 28.8% in Mtp-2.

**Conclusion:** The integration of the Aβ42/Aβ40 ratio in the PLM_R_ scale resulted in an easy-to-use tool which reduced the discrepancies in biologically doubtful cases and increased the confidence in the diagnosis in memory center.

## Background

Cerebrospinal fluid (CSF) protein biomarkers are nowadays included in guidelines of the National Institute of Aging-Alzheimer’s Association (NIA-AA) and the International Working Group 2 to diagnose Alzheimer’s disease (AD) in clinical research settings (McKhann et al., 2011; Dubois et al., 2014). CSF Aβ42, tau, and/or phosphorylated tau on Threonine 181 [ptau(181)] are validated to identify AD pathology in an early phase of the disease and for differential diagnosis (Andreasen et al., 2001; Engelborghs et al., 2008; Le Bastard et al., 2010; Gabelle et al., 2011; Schoonenboom et al., 2012; Marelli et al., 2015). Those biomarkers have been progressively integrated in daily clinical practice (Gabelle et al., 2013; Molinuevo et al., 2014; Mouton-Liger et al., 2014; Troussiere et al., 2014; Blennow et al., 2015; Lewczuk et al., 2015a; Paquet et al., 2016), however, the interpretation of results needs expertise and caution. In order to harmonize the clinical interpretation of the CSF biomarker profiles, the discovery of a new easy-to-use tool was an absolute requirement. Therefore, we developed the PLM (Paris-Lille-Montpellier) scale. This scale combines the concentration of the three CSF biomarkers [Aβ42, tau, ptau(181)] into a probability scale for AD (Lehmann et al., 2014). The score ranges from 0 to 3 based on the number of abnormal CSF biomarkers. The PLM scale outperformed CSF biomarkers when used alone, as compared to CSF ptau(181) which is known to be an efficient biomarker for discriminating AD from non-AD (NAD) patients (Vanderstichele et al., 2006; Gabelle et al., 2013; Struyfs et al., 2015). In addition, the predictive value of the PLM scale equalled those of logistic regression approaches, but with a better ranking as evaluated by calculation of the net reclassification index (NRI).

Since a few years, the relevance of the CSF Aβ42/40 ratio as a new biomarker has emerged as a method to minimize biases linked to pre-analytical or analytical factors (Nutu et al., 2013; Sauvee et al., 2014; Dorey et al., 2015; Lewczuk et al., 2015b), to improve the diagnostic performance of CSF biomarkers especially in discordant cases (Dumurgier et al., 2015) and for use in clinical routine (Lewczuk et al., 2004; Sauvee et al., 2014; Dorey et al., 2015). As the baseline level of Aβ peptides differs between individuals (Wiltfang et al., 2007), the ratio is also interesting to document a specific decrease in Aβ42 as an indication for on-going amyloidopathology. Recent studies comparing PET amyloid imaging and CSF biomarkers demonstrated that the Aβ42/40 ratio resulted in a better concordance than Aβ42 alone (Janelidze et al., 2016; Leuzy et al., 2016; Lewczuk et al., 2017). In addition, the fact that the Aβ ratio reflects an altered Aβ kinetics may allow an AD diagnosis before PET amyloid deposition is detectable (Patterson et al., 2015).

Thus, we aimed at evaluate if the outcome of the PLM scale could be improved by the integration of the Aβ42/40 ratio, instead of using Aβ42 alone. The new scale, named PLM_R_ scale was composed of four classes based on the results of the CSF Aβ42/40 ratio, tau and ptau(181) biomarkers. As the PLM scale, CSF results of a given patient are scored between 0 and 3 points. Score 0 corresponds to a normal profile [above cutoff for Aβ42/40 ratio, below cutoff for tau and ptau(181)]. Score 1 is related to one abnormal result in either Aβ42/40, tau or ptau(181). Score 2 corresponding to two pathologic values out of three biomarkers; and score 3 with all three being pathological. The new PLM_R_ scale was evaluated in a large monocentric study of AD and NAD patients. We compared the distribution of the AD and NAD, the percentage of discordant amyloid profiles and the diagnostic performance of the classic PLM scale and the PLM_R_ scale. We also took advantage of the opportunity to analyze two independent cohorts: one in which the ratio was quantified systematically; another one in which the ratio was determined only on biologically discordant cases.

## Materials and Methods

### Study Design and Participants

Participants with cognitive impairment were prospectively recruited and followed in the memory resources center of Montpellier (CMRR of Montpellier ^1^). Patients gave their informed and written consent to participate in the study and to have their samples stored in an officially registered and ethically approved biological collection (#DC-2008-417) at the Montpellier CHU’s certified NFS 96-900 biobank (Reference No. BB-0033-00031 ^2^). The authorization for handling personal data has been granted by the French Data Protection Authority (CNIL) under the number 1709743 v0. These participants were analyzed separately into two chronologically different cohorts: Mtp-1 (recruited from 07/2015 to 05/2017) and Mtp-2 (recruited from 09/2009 to 06/2015). These two timescales correspond to different approaches for using CSF Aβ40 and the CSF Aβ40/Aβ42. In the Mtp-2 cohort (the older one), the measurement of CSF Aβ40 was performed only in case of biological doubt, corresponding to (i) a value located in the gray-zone, which was defined as -1 SD (standard deviation) from the local cut-off value used, and/or (ii) a discordance between the clinical hypothesis and the value of CSF Aβ42 (in most cases; there was a strong clinical suspicion that patient had AD, while the CSF Aβ42 values were normal). In the Mtp-1 cohort (the more recent one), the measurement of CSF Aβ40 was performed systematically in routine, in a consecutive way. This approach has been put in place in parallel with the automation of the biomarker testing using the Euroimmun ELISAs in the Biological Lab.

For diagnostic purposes, all patients underwent a thorough clinical examination, including biological lab tests, neuropsychological evaluations, and brain imaging. Patients were classified into two groups: AD (as defined by the NINCDS-ADRDA criteria) (McKhann et al., 1984), and non-AD (NAD) patients. NAD diagnosis (e.g., fronto-temporal lobar degeneration, semantic dementia, Lewy body and Parkinson diseases, progressive supranuclear palsy, amyotrophic lateral sclerosis, normal pressure hydrocephalus, and psychiatric disorder) was defined by the commonly validated international criteria.

### CSF Samples and Assays

Cerebrospinal fluid was collected using standardized collection, centrifugation, and storage conditions (Del Campo et al., 2012; Dumurgier et al., 2013b). CSF tau and ptau(181) concentrations were measured using standardized commercially available INNOTEST_R_ sandwich ELISA according to the manufacturer’s procedures (Fujirebio Europe NV, Formely Innogenetics NV). We validated in our total population the optimal cutoffs of 400 and 60 pg/mL for tau and ptau(181), respectively (Lehmann et al., 2014). In the cohort Mtp-1, CSF Aβ1-42 and Aβ1-40 (named here Aβ42 and Aβ40) were measured with Euroimmun kits [EQ-6511-9601 (Aβ1-40); EQ-6521-9601 (Aβ1-42)]. The cut-off value for CSF Aβ42 was 500 pg/mL, while the cut-off for the Aβ42/40 ratio was 0.1. These values, which were validated in our cohort, have been initially proposed following a comparison between amyloid PET imaging ([18F]-flutemetamol PET) and CSF biomarkers (Palmqvist et al., 2015). In Mtp-2, CSF Aβ42, and Aβ40 were measured using INNOTEST_R_ sandwich ELISA according to the manufacturer’s procedures (Fujirebio Europe NV, Formely Innogenetics NV). The optimal cutoff for Aβ42 and Aβ42/40 ratio in our population was 700 and 0.05 pg/mL, respectively (Lehmann et al., 2014). The pre-analytical protocol was standardized (Del Campo et al., 2012) (same processing time, centrifugation, freeze/thaw cycle, aliquoting tubes) but differed between the two cohorts by the type of collection tubes used in each case (Perret-Liaudet et al., 2012b). Along with the fact that kits from different providers were used, this explained the difference in Aβ42 cut-off values. We did not assess the presence of an upward drift (Tijms et al., 2018) in our two cohorts that encompass only 3 to 4 years each. This could have improved the accuracy for both scales but was complicated by the fact that we have kits from two different venders.

As data have been generated through the routine activity of the laboratory, different lot numbers of each assay kit were used within the biological Lab. The quality of the results has been ensured by the use of validated standard operating procedure and internal quality controls (QC). The range of the QC coefficient of variation for the CSF analytes within each lot or across different lots was below 15%. In addition, the use of external QC ensured the quality of our results (Dumurgier et al., 2013b).

### Statistical Analysis

Statistical analyses were performed with the MedCalc software (17.6). Distribution of AD and NAD patients in the different PLM scales were computed and compared using ANOVA tests. Profiles with discrepancies among biomarkers (see legend) were also computed and compared. Receiver Operating Characteristic (ROC) curves were used to represent sensitivity and specificity for AD detection. ROC curves were generated from continuous diagnostic variables. The CSF biomarkers and their ratio are continuous. Regarding the scales, we plotted the curve using the four values 0, 1, 2, and 3 affected to the different samples.

To compare the classification of patients, we used the NRI (Pencina et al., 2008). The NRI is based on reclassification constructed separately for participants with and without the event of interest (i.e., AD or NAD diagnosis), and quantifies the correct movement into classes, upwards for events and downwards for non-events. At first, the following probabilities were calculated: p(up_AD) = (number of cases were the class was moving up between two classifications of AD patients)/(number of AD patients); p(down_AD) = (number of cases were the class was moving down between two classifications of AD patients)/(number of AD patients); p(up_NAD) = (number of cases were the class was moving up between two classifications of NAD patients)/(number of NAD patients); p(down_NAD) = (number of cases were the class was moving down between two classifications of NAD patients)/(number of NAD patients). We assumed that correctly classifying an AD patient was as important as correctly classifying a NAD patient and therefore we computed the NRI using the formula: NRI = (p(up_AD)-p(down_AD))-(p(up_NAD)-p(down_NAD)).

## Results

### Demographical and CSF Biomarkers Characteristics of Our Population (**Table 1**)

Nine hundred and four subjects were selected for the present study. The Mtp-1 cohort consisted of 400 patients (124 AD and 276 NAD), while 504 patients (218 AD and 286 NAD) were recruited into the Mtp-2 cohort. In the whole population, the mean age of the AD patients was 70.3 years (*SD*: ±9.2) and 66.5 (*SD*: ±12.1) for the NAD group of patients. Differences were found for age, MMSE and CSF biomarkers profile in the whole population and in each cohort. As expected, the AD patients were older (*p* < 0.0001), presented a lower MMSE score (*p* = 0.0002), a lower CSF Aβ42 concentration and higher CSF tau and ptau(181) levels (*p* < 0.0001 for all). For the AD patients in the Mtp-1 cohort, the mean CSF Aβ42 and CSF Aβ42/40 ratio was 553 ± 216 and 0.069 ± 0.022, respectively; and in the Mtp-2 cohort, the mean CSF Aβ42 and CSF Aβ42/40 ratio was 702 ± 335 and 0.045 ± 0.02, respectively. Concentrations of the biomarkers are affected by age for Aβ42 (*p* < 0.0001), tau (*p* = 0.0003), ptau(181) (*p* < 0.0001), and the Aβ42/40 ratio (*p* < 0.001) (*data not shown*).

**Table 1:** Demographical and CSF biomarkers characteristics on the whole population (n = 904) and for each cohort (Mtp-1 and Mtp-2).

	Whole population (*n* = 904)					Mtp-1 cohort (*n* = 400)					Mtp-2 cohort (*n* = 504)				
	NAD (*n* = 562)		AD (*n* = 342)			NAD (*n* = 276)		AD (*n* = 124)			NAD (*n* = 286)		AD (*n* = 218)		
	Mean	*SD*	Mean	*SD*	*p*	Mean	*SD*	Mean	*SD*	*p*	Mean	*SD*	Mean	*SD*	*p*
Age (years)	66.5	12.1	70.3	9.2	<0.0001	67.1	11.5	70.6	9.1	0.0031	66.0	12.6	70.1	9.3	0.0001
Sex (% male)	52.3		46.5		0.4077	53.3		38.7		0.2153	51.4		50.9		1.000
MMSE score	22.4	6.6	19.9	5.3	0.0002	23.4	4.5	20.1	4.8	0.0372	22.2	6.9	19.8	5.4	0.0011
CSF Aβ42	NA		NA			910	401	553	216	<0.0001	819	370	702	335	0.0003
CSF Aβ42/40	NA		NA			0.131	0.040	0.069	0.022	<0.0001	0.071	0.037	0.045	0.020	<0.0001
CSF tau	338	272	680	289	<0.0001	349	288	731	263	<0.0001	327	256	651	299	<0.0001
CSF ptau	43.2	21.8	90.0	34.5	<0.0001	43.2	20.7	94.4	33.4	<0.0001	43.2	22.9	87.5	34.9	<0.0001

*Results are expressed as the mean ± standard deviation (SD). The difference between the two groups NAD and AD was determined by a *t*-test. AD, Alzheimer disease; NAD, non-Alzheimer disease; NA, not analyzed in this cohort; CSF, cerebrospinal fluid; MMSE, mini-mental scale examination; Mtp, Montpellier.*

### Distribution of AD and NAD Between the PLM Classes (**Figure 1**)

**FIGURE 1:**
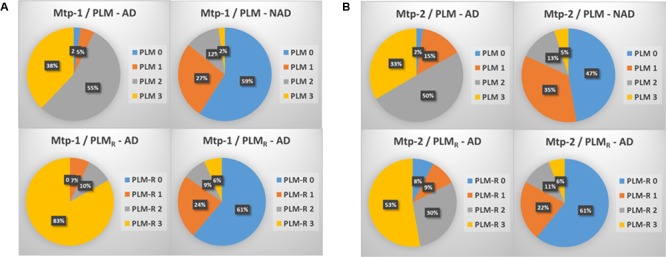
The distribution in percentage of AD and NAD patients in the two cohorts (**A**: MTP-1 and **B**: MTP-2) when classified using the PLM-scale or the PLM_R_ scale. Note important shift in the distribution of the AD patients between the two scales (ANOVA, *p* < 0.0001, PLM-AD vs. PLM_R_-AD).

The distribution of the patients in PLM classes within the two cohorts, generated in a consecutive way or after selection of the discordant/borderline profiles, was slightly different (**Figures 1A,B**). The main difference was a higher percentage of AD patients in class 1 (15 vs. 5%), and a higher percentage of NAD in class 2 (35 vs. 27%) in the Mtp-2 cohort. When PLM and PLM_R_ scale distributions were compared, the difference in both cohorts was highly significant (ANOVA, *p* < 0.0001). The most dramatic modification for PLM_R_ was an increase of the percentage AD in class 3 from 38 to 83% in Mpt-1 and from 33 to 53% in Mpt-2 cohort. This was accompanied by a parallel decrease of the percentage of AD in class 2, from 55 to 10% in Mtp-1 and from 50 to 30% in Mpt-2. The distribution within the NAD group was also modified, especially in the Mtp-2 cohort going from 47% in the class 0 with PLM to 61% with PLM_R_.

### Percentage of Discordant Amyloid Profiles in PLM and PLM_R_ Scales (**Table 2**)

Discrepant results for the CSF AD Aβ biomarkers correspond to two situations: (1) normal values for Aβ42 or Aβ42/40 combined with pathological values of tau and ptau(181). These cases are included in class 2 (termed PLM2-Aβ42 and PLM_R_2-Ratio); (2) pathological values of Aβ42 or Aβ42/40 combined with normal values of tau and ptau(181) (termed PLM1-Aβ42 and PLM_R_1-Ratio). The use of the Aβ42/40 ratio was marked by a sharp reduction of the discordant profiles going from 34 and 37.5% for the PLM scale, to 16.3 and 19.8% with the PLM_R_, for the Mtp-1 and Mtp-2 cohort, respectively. We noticed that in the cohort Mtp-2, the impact of using the PLM_R_ is important for both types of discrepancies, while for Mtp-1, it was restricted to PLM1-Aβ42. Of note, we do not expect to reduce the discrepancy to zero as there are clinical situations with amyloidogenic profile without alteration of tau, and conversely.

**Table 2:** Discrepancies of PLM scale and PLM_R_ scale: the discordant profiles linked to CSF amyloid biomarkers.

Cohort	PLM discrepancy	PLM2-Aβ42	PLM1-Aβ42	PLM_R_ discrepancy	PLM_R_2-ratio	PLM1-ratio
Mtp-1	34.0%	22.8%	11.3%	16.3%	5.8%	10.5%
Mtp-2	37.5%	20.8%	16.7%	19.8%	13.3%	6.5%

*Discordant results correspond to two different situations. Firstly, when values of Aβ42 or Aβ42/40 were normal while tau and ptau were pathologic (named her as PLM2-Aβ42 or PLM_*R*_2-Ratio). Secondly, when values of Aβ42 or Aβ42/40 were pathologic, while tau and ptau were normal (named here as PLM1-Aβ42 or PLM_*R*_1-Ratio). These values in percentage of the sample with the profile are reported on the table. The sum of the two types of discrepancies is computed for both the PLM and the PLM_*R*_ scale in the two cohorts.*

### Diagnostic Performance of the PLM and PLM_R_ Scales (**Figure 2** and Supplementary Tables 1–3)

**FIGURE 2:**
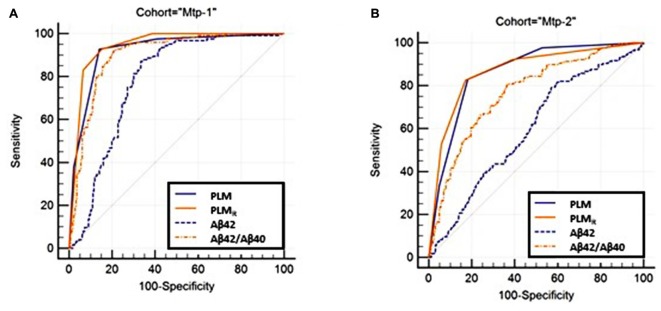
The ROC curve of the PLM, PLM_R_-scales and CSF Aβ42, Aβ42/40 ratio are plotted (Supplementary Table 1). Statistical analysis (Supplementary Table 2) confirmed that the PLM_R_ outperformed all the other situations in both cohorts. **(A)** MTP-1 and **(B)** MTP-2.

The diagnostic performance of the PLM and PLM_R_ scales was compared firstly by ROC analysis (**Figure 2**). The AUC of the PLM_R_ scale was 0.94 in the Mtp-1 cohort, and 0.87 in the Mtp-2 cohort (Supplementary Table 1). In both cohorts, the PLM_R_ outperformed CSF Aβ42 (*p* < 0.0001) or the CSF Aβ42/40 ratio (*p* < 0.0001 for Mtp-2 and *p* = 0.03 for Mtp-1). The PLM_R_ has an AUC significantly higher than that of PLM only for the Mtp-1 cohort (Supplementary Table 2). The higher discriminatory values in the Mpt-1 cohort relates, we believe, to the fact that in this case Aβ40 was performed systematically and not only in discordant cases.

Secondly, we compared the diagnostic performance of both scales by calculation of the NRI (Supplementary Table 3). With an NRI equal to 44.3% in the Mtp-1, and 28.8% in the Mtp-2, the PLM_R_ improved the classification of AD or NAD patients and the confidence of the diagnosis. Looking carefully at the NRI data, it appeared that in the Mtp-1 cohort, the improvement linked to PLM_R_ was mainly significant for AD patients (“up if AD”), while in Mtp-2, it was both for AD and NAD (“down if NAD”) patients.

## Discussion

By integrating the CSF Aβ42/40 ratio in the PLM scale, we observed that the number of discordant biomarker profiles have been significantly reduced. Our findings have highlighted the interest of the use of the CSF Aβ42/40 ratio, as it can reduce pre-analytical bias (Perret-Liaudet et al., 2012a,b; Vanderstichele et al., 2016) and might help the interpretation of protein biomarker profiles in the context of screening for the presence of amyloidopathies (Palmqvist et al., 2015; Lewczuk et al., 2017). We also demonstrated that the combination of CSF biomarkers within the PLM scale (Lehmann et al., 2014) or the PLM_R_ scale presents an added value in terms of clinical performance and usability. Importantly, the PLM_R_ scale is more relevant than the classical PLM scale and it improved the confidence toward the AD diagnosis as confirmed by the NRI computation in both cohorts. Interestingly, the accuracy of PLM_R_ (as measured by the AUC) is increased only in the Mtp-1 cohort. The reason might be that in this cohort, Aβ40 was analyzed systematically in all samples and not only in discordant cases as in the Mtp-2 cohort.

One major impact was the ability to compare the scales in two large cohorts differing in terms of the use of the Aβ42/40 ratio. This approach was implemented in our Biological Lab along with the automation of the ELISA assays using the Euroimmun CE IVD kits for Aβ42 and Aβ40. The automation and parallel measurement of the two analytes reduced variability when compared to manual measurement (Chiasserini et al., 2016). It also facilitates, together with the code bar readout lecture of components and internal controls, the accreditation of these measurements under the quality norm ISO15189 (not shown). This prefigures the future use of random access automatized analyzers that will even further reduce variabilities. In addition, the measurement in consecutive samples ameliorates the turnover and reduces delay of results of the Biological Lab when compared to a situation where the Aβ40 measurement is performed only in some discordant or specific cases. That was the approach used in the Biological Lab previously. These two procedures have generated series of samples that differ, as illustrated by the number and types of discordant profiles mostly in the Mtp-2 cohort. The performance of the Aβ42 biomarker was also logically lower in this cohort and conversely, the impact of the Aβ42/40 ratio was more important. In some studies, the approach to use the Aβ42/40 ratio only in some cases was preferred (Dumurgier et al., 2015). Anyway, the purpose of our study was not to compare both approaches that used by the way in our case different analytical kits for Aβ42 and Aβ40 measurement. It was, however, important to evaluate the interest of integrating the Aβ42/40 ratio into the PLM scale in the two contexts. It thus appeared that the PLM_R_ scale was an easy-to-use tool in clinical practice without the need of hard calculation same as the logistic regression model developed by Spies et al. (2013). Based on early phase of AD, the Erlangen scale seemed also interesting; however, the large number of classes referred to different possibilities of biomarker combinations was less intuitive than the PLM scale. The concentrations of the biomarkers are affected by age for Aβ42, tau, ptau(181), and the Aβ42/40 ratio. This finding is in accordance of previous results from the PLM group (Dumurgier et al., 2013a) and others (Mattsson et al., 2012).

The strengths of our study were the large number of participants and the standardized procedure for clinical diagnosis and for the dosage of the CSF biomarkers in the same biological Lab. Despite the differences of the two cohorts, they mimic well the current situation were clinical laboratory are either doing the measurement of CSF Aβ40 systematically or only in some biologically doubtful cases. The present study has however, some limitations. We do not have neuropathological confirmation cases to validate the AD and NAD diagnosis. However, as a monocentric study, the diagnosis of AD and NAD patients have been determined based on international diagnosis criteria and accordingly to an expert’s consensus within the memory center. It will be interesting also to integrate the APOE𝜀4 status in the analysis; however, as the study was performed in a clinical setting, such assessment is only available for a small number of participants that not give us enough statistical power to implement this variable in our model. Further studies were ongoing to integrate the APOE𝜀4 status in the PLM scale especially for early cases of AD or at-risk to develop AD participants. No PET amyloid was available in clinical settings but furthers studies need to be done to determine the relevance of the PLM_R_ scale concerning the discordant cases between CSF Aβ42 and PET amyloid load results.

## Conclusion

We proposed an optimized scale, the PLM_R_ scale, that integrated the Aβ42/40 ratio and allowed us to better define AD patients in clinical routine in a memory center. Further studies are needed to investigate if the PLM_R_ scale could be used also for discrimination of different AD subtypes. This easy-to-use tool reduced the discrepancies in biologically doubtful cases and increased the confidence of the diagnosis. It is also compatible with multiple cutoffs resulting from different preanalytical protocols and kit providers, and it is well-adapted to the future improvement of the AD biomarkers assays that will benefit from fully automatized systems.

## Author Contributions

SL: data analysis and interpretation, statistical analysis, study concept, and drafting/revising the manuscript. CD, LT, and SN: data acquisition and revising the manuscript. GB, CC, NG, and AM: data acquisition. CP and JD: revising the manuscript. EV and HV: data interpretation and drafting/revising the manuscript. AG: data analysis and interpretation, study concept, study supervision, and drafting/revising the manuscript.

## Conflict of Interest Statement

EV and HV are co-founders of ADx NeuroSciences. In this study, these personals were not directly involved in the setting or the design of the experiments. They however provided us scientific background information and ELISA kit free of charge. The other authors declare that the research was conducted in the absence of any commercial or financial relationships that could be construed as a potential conflict of interest.

## Footnotes

1 www.chu-montpellier.fr/fr/cmrr/

2 www.biobanques.eu
