# Supplementary material for: Relevance of Aβ42/40 Ratio for Detection of Alzheimer Disease Pathology in Clinical Routine: The PLMR Scale
Source: Front Aging Neurosci. 2018 May 28;10:138. doi: 10.3389/fnagi.2018.00138 (PMC5985301; doi:10.3389/fnagi.2018.00138)
Supplement: Supplementary file 1 [file Data_Sheet_1.docx]

**SUP TABLES**

## Sup Table 1: ROC analysis (AUC) for differentiation of AD from NAD using the PLM scale, PLM_R_-scale, CSF Aβ42 and CSF Aβ42/40 ratio in the two study cohorts.

|  | **Mtp-1 cohort** | | |  | **Mtp-2 cohort** | | |  | |
| --- | --- | --- | --- | --- | --- | --- | --- | --- | --- |
|  | **AUC** | **SE** | **95% CI** |  | **AUC** | **SE** | **95% CI** | |  |
| **PLM** | **0.92** | **0.014** | **0.890 - 0.945** |  | **0.86** | **0.016** | **0.832 - 0.894** | |  |
| **PLM_R_** | **0.94** | **0.010** | **0.915 - 0.963** |  | **0.87** | **0.016** | **0.836 - 0.897** | |  |
| **CSF Aβ42** | **0.78** | **0.023** | **0.734 - 0.818** |  | **0.60** | **0.025** | **0.553 - 0.641** | |  |
| **CSF Aβ42/40** | **0.90** | **0.016** | **0.865 - 0.926** |  | **0.77** | **0.021** | **0.728 - 0.803** | |  |
| **CSF Tau** | **0.87** | **0.018** | **0.835 - 0.903** |  | **0.84** | **0.017** | **0.808 - 0.874** | |  |
| **CSF pTau** | **0.94** | **0.012** | **0.921 - 0.968** |  | **0.89** | **0.014** | **0.867 – 0.922** | |  |

## Abbreviations: AUC = area under the curve; CI = confidence interval; SE = standard error

## Sup Table 2: Statistical comparison (DeLong) between the AUC of the different ROC curves.

|  | **Mtp-1 cohort** |  | **Mtp-2 cohort** | **Both cohorts** |
| --- | --- | --- | --- | --- |
|  | **Significance level** |  | **Significance level** | **Significance level** |
| **PLM_R_ vs PLM** | **P = 0.009** |  | **P = 0.69** | **P = 0.69** |
| **PLM_R_ vs CSF Aβ42** | **P < 0.0001** |  | **P < 0.0001** | **NA** |
| **PLM_R_ vs CSF Aβ42/40** | **P = 0.003** |  | **P < 0.0001** | **NA** |
| **PLM vs CSF Aβ42** | **P < 0.0001** |  | **P < 0.0001** | **NA** |
| **PLM vs CSF Aβ42/40** | **P = 0.211** |  | **P < 0.0001** |  |
| **CSF Aβ42 vs CSF Aβ42/40** | **P < 0.0001** |  | **P < 0.0001** | **NA** |

## Abbreviations: NA not applicable (since the cutoffs of Aβ42 and Aβ42/40 are not the same in the two cohorts)Sup Table 3:

To compute the NRI between the PLM and the PLM_R_ classification, the modification in the value of the scales is followed in the AD and the NAD situations. The percentage of samples in each situation is reported on the table. As illustrated, the major impact of the PLM_R_ scale is to improve the classification of AD patients and the confidence of the diagnosis.

| **Cohort** | **UP if AD** | **DOWN if AD** | **UP if NAD** | **DOWN if NAD** | **NRI (PLM🡪PLM_R_)** |
| --- | --- | --- | --- | --- | --- |
| **Mtp-1** | **48.4%** | **0.8%** | **8.0%** | **4.7%** | **44.3%** |
| **Mtp-2** | **32.5%** | **15.4%** | **7.5%** | **19.1%** | **28.8%** |
